# New strategy of a lung compensating technique with STR for total body irradiation

**DOI:** 10.1002/acm2.13791

**Published:** 2022-10-05

**Authors:** Yuya Yanagi, Hajime Monzen, Mikoto Tamura, Masakazu Otsuka, Yasumasa Nishimura

**Affiliations:** ^1^ Department of Medical Physics, Graduate School of Medical Sciences, Kindai University Osakasayama Osaka Japan; ^2^ Department of Radiology, Shiga University of Medical Science Hospital Otsu Shiga Japan; ^3^ Department of Radiology Center, Kindai University Hospital Osakasayama Osaka Japan; ^4^ Department of Radiation Oncology, Faculty of Medicine, Kindai University Osakasayama Osaka Japan

**Keywords:** lung compensating filter, soft variable shape tungsten rubber (STR), total body irradiation (TBI), water‐equivalent dose

## Abstract

**Purpose:**

To determine the thickness of a soft variable shape tungsten rubber (STR) as a lung compensating filter in total body irradiation.

**Methods:**

A tough water (TW) phantom and tough lung (TL) phantom were used as water and lung‐equivalent phantoms. The TW with a thickness of 3 cm simulating the thoracic wall was used (upper layer). The TW or TL with a thickness from 1 to 15 cm (1 cm increments) was placed beneath the upper layer (middle layer). The TW with a thickness of 5 cm simulating the mediastinum was placed beneath the middle layer (lower layer), and a farmer ionization chamber was placed beneath this layer. The relative doses of a 10 MV X‐rays were then measured. The TL was compensated in 1 mm increments from 1 to 11 mm of the STR, and the thickness of the STR at the same dose of TW (water equivalent) was obtained.

**Results:**

The compensating ability of STR increased as the thickness of the TL increased, and an STR with a thickness of 1 mm reduced the dose by 2%–4%, depending on the thickness of lung. The STR thickness as an equivalent dose of TW per cm of TL was approximately linear, and the thickness was 0.62 mm/cm of TL.

**Conclusion:**

The STR can be used as a lung compensating filter for a water equivalent dose with 0.62 mm of STR per cm of lung.

## 1 INTRODUCTION

Total body irradiation (TBI) is used before hematopoietic stem cell transplants for leukemia to restrain immunity and control tumors.^1^, ^2^ Although the target volume of TBI is the whole body, dose differences occur at various locations because the body thickness and density are heterogeneous. Thus, a patient's whole body dose is permitted to be within ±5%–10% of the prescribed dose.^3^, ^4^ The expanded source surface distance (SSD) technique has been widely used because it does not require an additional cost, and it is easy to install in general purpose radiation treatment equipment. In regard to the patient's position, a bilateral position with horizontal beams (i.e., supine position) is stable and has high reproducibility.^1^ According to surveys of various countries by Ishibashi et al., Koken et al., and Giebel et al., the TBI techniques, including patient position, prescribed dose, and protection methods of organs at risk (OAR), vary significantly and have not converged in the past 30 years.^5^, ^6^, ^7^

Pulmonary fibrosis and interstitial pneumonitis are acute and late toxicities of the lung, and their incidence is related to the TBI technique.^8^, ^9^ In the case of the supine position, positioning the patients’ arms beside their thorax can reduce excess dose to the lung during TBI^10^; however, the shielding volume is insufficient when only the patients’ arms are used. Acrylic or lead has also been used to shield OARs; however, a large amount of acrylic is necessary because of its density, and lead is toxic.^10^, ^11^ In addition, the placement of the compensating materials is a problem in the expanded SSD technique.

The previous studies have reported electron beams, γ‐rays, and X‐rays shielding abilities of tungsten functional paper and tungsten‐containing rubber and investigated their applications.^12^, ^13^, ^14^, ^15^, ^16^, ^17^, ^18^, ^19^, ^20^ The other studies have also developed a real time soft variable shape tungsten rubber (STR) (Hayakawa Rubber Co., Ltd., Hiroshima, Japan) and reported its radiation shielding ability.^21^, ^22^ The STR is reusable and can be freely formed by warming. Therefore, the STR can be attached to a patient to form the compensating material, with a high reproducibility of the placement position. As a result, the STR is superior to lead regarding both cost and environment. This study has three aims: to characterize the shielding ability of the STR, evaluate the lung compensation ability of the STR, and comment on the placement of the STR for clinical use.

## 2 MATERIALS AND METHODS

X‐rays were delivered using a 10 MV photon beam on a Novalis Tx linear accelerator (Varian Medical Systems, Palo Alto, CA, USA). Each measurement was performed using a water‐equivalent phantom (tough water; TW) (physical density; 1.017 g/cm^3^) or lung‐equivalent phantom (tough lung; TL) (physical density; 0.320 g/cm^3^) (Kyoto Kagaku Co., Ltd., Kyoto, Japan) with a farmer ionization chamber Type 30013 (PTW, Freiburg, Germany). The phantom dimensions were 300 mm × 300 mm × each thickness. The electrometer RAMTEC Smart (Toyo Medic, Tokyo, Japan) was used, and the bias voltage was set to −300 V. The relative dose measurements were corrected for temperature and air pressure to compensate for the respective measurement conditions.

### 2.1 Evaluation of the radiation quality by distance

**FIGURE 1:**
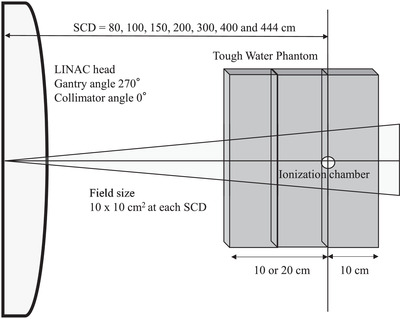
Schematic of the setup used to evaluate the radiation quality by the source‐to‐chamber distance (SCD)

To evaluate the radiation quality at different distances, the tissue phantom ratio (TPR_20,10_) was measured at different sources to chamber distances (SCDs). TW and an ionization chamber were used at a 270° gantry angle and 0° collimator angle. The SCD was set to 80, 100, 150, 200, 300, 400, and 444 cm. The clinical distance in our institution is 444 cm. Each angle notation conformed to IEC 61217.^23^ The irradiated field was 10 × 10 cm^2^ at each SCD. Ten centimeters of TW were behind the ionization chamber, and 10 or 20 cm of TW was in front of the ionization chamber. TPR_20,10_ was calculated by comparing the relative dose between 10 and 20 cm of TW placed in front of the chamber. Figure 1 shows the setup.

### 2.2 Characteristics of STR

The STR containing powdered tungsten acts as a radiation shield, while maintaining the characteristics of rubber. It is possible to change the shape of STR at 60–70°C, and the STR maintains its shape at room temperature. The elemental ratio (wt%) in STR with a density of 7.3 g/cm^3^ was C: 5.5%, H: 0.9%, O: 1.4%, and W: 92.2%.^21^

### 2.3 Evaluation of the shielding ability of STR

**FIGURE 2:**
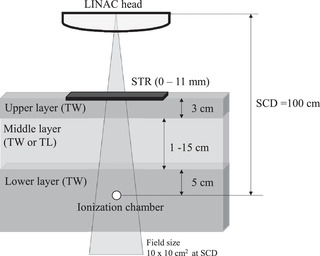
Schematic of the setup used to evaluate the shielding and lung compensation of the soft variable shape tungsten rubber (STR). Upper and lower layer consisting of tough water (TW). Middle layer consisting of either TW or tough lung (TL). The STR was placed on the surface of the upper layer.

In this study, the TW and TL were used to evaluate the shielding ability of STR. Figure 2 shows the setup. The gantry and collimator angles were 0°, and the irradiated field was 10 × 10 cm^2^ at SCD = 100 cm. First, 3 cm of TW simulating the thoracic wall was placed (upper layer). Then, the TL was placed in 1 cm increments, from 1 to 15 cm, beneath the upper layer (middle layer). Finally, 5 cm of TW simulating the mediastinum was placed beneath the middle layer (lower layer), and an ionization chamber was positioned beneath this layer. The STR changing by 1 mm from 1 to 11 mm was placed on the surface of the upper layer, and the relative dose was measured in each thickness of TL. The dose with an STR of thickness of 0 (i.e., without STR) was normalized to 100%, and the relative dose with each thickness of TL and STR was evaluated.

### 2.4 Evaluation of the lung compensating ability with STR

The TW and TL were used to evaluate the lung compensating ability with an STR. The irradiation conditions were the same as the shielding ability of STR. The TL of the middle layer was replaced with TW, and the lung compensating rate was defined as the relative dose of TL with STR compared with the dose of the same thickness of TW normalized to 100%. Eq.STR was defined as the STR thickness that could create an equivalent dose to the thickness of TW at each TL thickness. Eq.STR was calculated from a linear approximation when STR is varied from 1 to 11 mm for each TL thickness.

## 3 RESULTS

### 3.1 Evaluation of the radiation quality by distance

Table 1 shows the TPR_20,10_ at each SCD. The TPR_20,10_ at each SCD was 0.739–0.745, the coefficient of determination (*R* ^2^) was 0.6099, and there was no strong correlation of SCD and TPR_20,10_, indicating no significant change in the radiation quality at each SCD.

**TABLE 1:** The tissue phantom ratio (TPR_20,10_) at each source‐to‐chamber distance (SCD)

TPR_20,10_ at each SCD	
SCD (cm)	TPR_20,10_
80	0.739
100	0.740
150	0.744
200	0.743
300	0.744
400	0.745
444	0.744
*R* ^2^	0.6099

*Note*: The TPR_20,10_ at each SCD was 0.739–0.745, the coefficient of determination (*R* ^2^) was 0.6099, and there was no strong correlation of SCD and TPR_20,10_.

### 3.2 Evaluation of the shielding ability of STR

Table 2 shows the transmission rate of STR, which is the relative dose of the TL compared with the dose of TL without STR normalized to 100%. The lateral direction indicates changing the TL thickness, and the vertical direction indicates the STR thickness. The gradient described the rate of attenuation per mm of STR at each TL thickness. The STR with a thickness of 1.0 mm reduced the dose by 2%–4%. The transmission rate of STR indicated a linear decrease whenever the TL thickness increased, the rate decreased 0.072% per mm of STR by increasing the TL by 1 cm.

**TABLE 2:** The transmission rate of soft variable shape tungsten rubber (STR)

		The transmission rate of STR														
		Transmission rate (%)														
		TL														
		1 cm	2 cm	3 cm	4 cm	5 cm	6 cm	7 cm	8 cm	9 cm	10 cm	11 cm	12 cm	13 cm	14 cm	15 cm
STR	0 mm	100.0														
	1 mm	97.3	97.2	97.2	97.2	97.1	97.0	97.1	97.0	97.0	97.0	97.0	96.9	97.0	96.9	97.0
	2 mm	94.9	94.9	94.8	94.7	94.6	94.5	94.5	94.5	94.4	94.4	94.4	94.3	94.4	94.3	94.3
	3 mm	92.3	92.3	92.2	92.0	91.9	91.8	91.7	91.7	91.6	91.6	91.5	91.5	91.5	91.4	91.5
	4 mm	89.9	89.7	89.6	89.4	89.3	89.2	89.1	89.0	88.9	88.9	88.8	88.8	88.7	88.7	88.6
	5 mm	87.5	87.3	87.1	87.2	86.8	86.6	86.8	86.5	86.3	86.2	86.2	86.1	86.1	86.0	86.0
	6 mm	85.1	84.9	84.7	84.5	84.3	84.1	84.0	84.0	83.7	83.7	83.6	83.6	83.5	83.4	83.4
	7 mm	82.9	82.6	82.4	82.1	81.9	81.7	81.6	81.5	81.4	81.2	81.2	81.0	81.1	80.9	80.9
	8 mm	80.6	80.4	80.1	79.8	79.6	79.4	79.2	79.1	78.9	78.9	78.7	78.6	78.6	78.5	78.4
	9 mm	78.5	78.2	77.9	77.6	77.4	77.1	77.0	76.8	76.6	76.5	76.4	76.3	76.3	76.1	76.1
	10 mm	76.6	76.2	76.0	75.7	75.4	75.2	75.0	74.9	74.6	74.6	74.4	74.3	74.2	74.1	74.0
	11 mm	74.5	74.2	73.9	73.6	73.3	73.0	72.8	72.7	72.4	72.4	72.2	72.1	72.0	71.9	71.8
Gradient (%)		−2.37	−2.45	−2.52	−2.59	−2.67	−2.72	−2.80	−2.87	−2.95	−3.00	−3.08	−3.16	−3.24	−3.31	−3.39

*Note*: The transmission rate of STR was the relative dose of the tough lung (TL) compared with the dose of TL without STR normalized to 100%. The lateral direction indicates changing the TL thickness, and the vertical direction indicates the STR thickness. The gradient described the rate of attenuation per mm of STR at each TL thickness.

### 3.3 Evaluation of the lung compensating ability with STR

**FIGURE 3:**
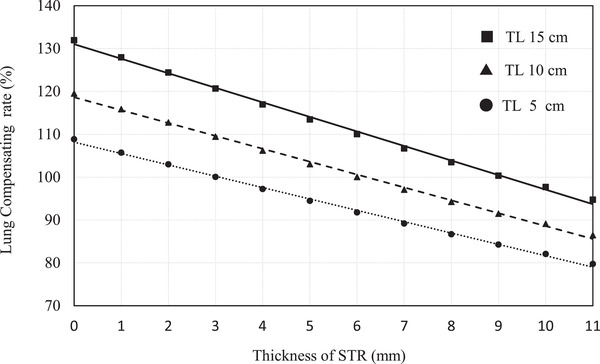
The compensating rates for tough lung (TL) with the thicknesses of 5, 10, and 15 cm using soft variable shape tungsten rubber (STR) with 1 mm increments over 1–11 mm.

**FIGURE 4:**
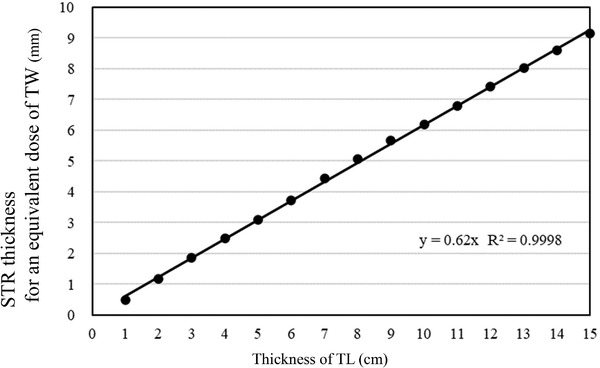
The relationship between the thickness of the tough lung (TL) and the thickness of the soft variable shape tungsten rubber (STR) required to make it water equivalent. The STR thickness calculated from the Eq.STR for each TL thickness was 0.62 mm.

Table 3 shows the lung compensating rate with STR. The lung compensating rate is defined as the relative dose of TL with STR compared with the dose of the same thickness of TW normalized to 100%. The thickness of STR that could be made equivalent dose to the thickness of TW at each TL thickness was obtained by linear interpolation and was expressed as Eq.STR. For example, Figure 3 shows the lung compensating rate of TL with a thickness of 5, 10, and 15 cm with STR changing in 1 mm increments over 1–11 mm. The lung compensating rate by the STR thickness was approximately linear for all TL thicknesses, and the coefficient of correlation was 0.9980, 0.9975, and 0.9976, at 5, 10, and 15 cm TL, respectively. The gradient of the attenuation rate per mm of STR became slightly stronger with −2.65, −3.00, and −3.39, respectively, as the TL thickness increased. The required thicknesses of STR per cm of TL for an equivalent dose of the same TW thickness were similar, and the STR thickness calculated from the Eq.STR for each TL thickness was 0.62 mm. Although the lung compensating ability of the STR slightly increases linearly with increasing TL thickness, the STR thickness for an equivalent dose of TW may be approximately linear. The relationship between the thickness of the TL and the thickness of the STR required to make it water equivalent was shown in Figure 4.

**TABLE 3:** The lung compensating rate of soft variable shape tungsten rubber (STR)

		The lung compensating rate of STR														
		Lung compensating rate (%)														
		TL														
		1 cm	2 cm	3 cm	4 cm	5 cm	6 cm	7 cm	8 cm	9 cm	10 cm	11 cm	12 cm	13 cm	14 cm	15 cm
STR	0 mm	101.7	103.4	105.2	107.0	108.9	110.9	113.1	115.3	117.5	119.5	121.8	124.3	126.8	129.4	131.9
	1 mm	98.9	100.5	102.2	103.9	105.7	107.7	109.8	111.9	114.0	115.9	118.2	120.5	123.1	125.4	127.9
	2 mm	96.5	98.1	99.7	101.3	103.0	104.8	106.9	109.0	110.9	112.8	115.0	117.3	119.7	122.0	124.4
	3 mm	93.9	95.4	97.0	98.5	100.1	101.8	103.8	105.8	107.7	109.5	111.5	113.7	116.0	118.3	120.7
	4 mm	91.3	92.8	94.2	95.7	97.2	98.9	100.8	102.6	104.5	106.2	108.2	110.4	112.6	114.7	117.0
	5 mm	88.9	90.3	91.6	93.3	94.5	96.1	98.2	99.7	101.4	103.0	105.0	107.0	109.2	111.3	113.5
	6 mm	86.5	87.8	89.1	90.4	91.8	93.3	95.1	96.8	98.4	100.0	101.9	103.9	105.9	107.9	110.0
	7 mm	84.2	85.4	86.7	87.9	89.2	90.7	92.4	94.0	95.6	97.1	98.9	100.7	102.8	104.7	106.7
	8 mm	81.9	83.1	84.3	85.4	86.7	88.1	89.6	91.3	92.8	94.2	95.9	97.8	99.7	101.6	103.5
	9 mm	79.8	80.8	81.9	83.0	84.3	85.6	87.1	88.6	90.1	91.5	93.1	94.9	96.7	98.5	100.4
	10 mm	77.8	78.8	79.9	81.0	82.1	83.4	84.8	86.4	87.7	89.1	90.6	92.4	94.1	95.9	97.7
	11 mm	75.7	76.7	77.7	78.7	79.7	81.0	82.4	83.8	85.1	86.5	87.9	89.6	91.3	93.0	94.7
Eq.STR (mm)		0.50	1.19	1.87	2.50	3.11	3.73	4.43	5.07	5.66	6.20	6.80	7.41	8.02	8.59	9.14

*Note*: The lung compensating rate was defined as the relative dose of tough lung (TL) with STR compared with the dose of the same thickness of tough water (TW) normalized to 100%. Eq.STR expressed the thickness of STR that could create an equivalent dose to the thickness of TW at each TL thickness.

## 4 DISCUSSION

We investigated the lung compensation by STR in TBI. The STR can be used to linearly compensate for the lung depending on the thickness of the lung. In addition, we confirmed the radiation quality did not depend on SCD. Thus, the inspection at SCD = 100 cm could be adjusted through SCD = 444 cm (Table 1).

The transmission rate per mm of STR linearly decreased with the TL thickness. In addition, the transmission rate per mm of STR slightly decreased by 0.072% when the TL thickness increased every 1 cm. Fujimoto et al. studied dosimetric shielding with tungsten in electron beams and reported tungsten generated scattered, transmission, and bremsstrahlung radiation.^12^ The same phenomenon might be caused in the photon beam case. As a result, a photon beam arriving at a measurement point gradually decreases because of the distance of the TL to the chamber.

The influence of the increase in STR shielding with increasing the TL thickness was not affected, as shown in Table 3 and Figure 4; the STR thickness necessary for an equivalent dose to TW per cm of TL was approximately linear. The necessary STR thickness for an equivalent dose to TW was 0.62 mm/cm of TL. A compensating table for lung using the STR was created. If other compensating materials are used, the compensating dose is presumed, and there is high versatility with measuring several points to acquire the correction formula calculated from the ratio of the attenuation rate of the compensating materials at different facilities. Furthermore, the table may lead to the standardization of the compensating method. Current TBI techniques are not standardized, and they vary, depending on the facility.^5^, ^6^, ^7^ Various techniques for OAR compensation have been used. In this study, we clarified the relation between the thickness of the lung and compensation with STR. For example, if the patient has a lung thickness of 10 cm, the thickness of STR as a water‐equivalent dose is 10 cm × 0.62 mm = 6.2 mm, or as a 70% water dose, it is 6.2 mm × 0.7 = 4.3 mm. As a result, the prescribed dose can easily be estimated from our data table.

**FIGURE 5:**
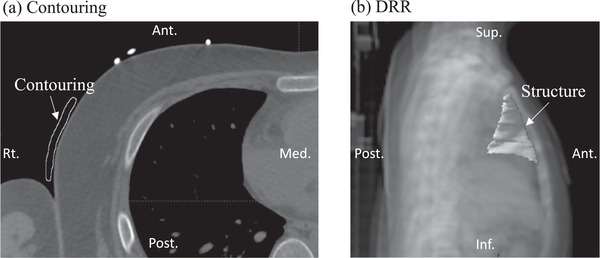
Contouring of compensating material in a treatment planning system (TPS) and digitally reconstructed radiograph (DRR) with soft variable shape tungsten rubber (STR). (a) Contouring of compensating material in TPS and (b) DRR with STR

The STR can be attached to the patient's skin because it can be shaped in real time and has no toxicity. TBI therapy is performed with multiple fractionated irradiations, and reducing the preparation time is important. With STR as a lung compensating filter in TBI, the preparation time after the second fraction can be greatly reduced by confirming the initial fraction with linacgraphy, processing in real time, and marking the patient's skin. Additionally, it may be possible to adjust without linacgraphy at every irradiation. Linacgraphy is MV image acquisition procedure. Mekdash et al. reported a simple technique using a field designed with a multi‐leaf collimator aperture conforming to lung contours using computed tomography (CT) simulations and a treatment planning system (TPS) to represent the lung that was subsequently marked on the patient's skin before TBI therapy.^24^ Although Mekdash et al. marked directly on the patient's skin, the shape of STR can easily be made in real time by projecting the irradiation field of the lung made using a CT simulator. In addition, the size and shape of the compensating materials can be estimated and easily used by contouring compensating materials in a required area in a TPS and creating the digitally reconstructed radiograph, as indicated in Figure 5. If such preparation is possible before TBI therapy, the preparation time is reduced, and the patient time and facility throughput can be optimized.

## 5 CONCLUSIONS

An STR filter thickness of 0.62 mm was required for every 1 cm of lung thickness when attached to a patient's skin. We determined the shielding ability and thickness of the STR to convert from lung to water and established a very simple compensating method in TBI.

## FUNDING

This work was supported partly by Japan Society for the Promotion of Science (JSPS) KAKENHI grant number 19K08211.

## DECLARATION OF COMPETING INTEREST

Hajime Monzen received a research donation from Hayakawa Rubber Co., Ltd.

## AUTHOR CONTRIBUTIONS

Yuya Yanagi and Hajime Monzen were associated with concept and design. Yuya Yanagi and Masakazu Otsuka took the measurements. Yuya Yanagi, Masakazu Otsuka, and Mikoto Tamura analyzed the data. Yuya Yanagi, Masakazu Otsuka, Hajime Monzen, Mikoto Tamura, and Yasumasa Nishimura prepared the manuscript. All authors read and approved the final manuscript.
